# Protective mucosal and systemic immunity induced by virus-like particles expressing *Toxoplasma gondii* cyst wall protein

**DOI:** 10.1371/journal.pone.0283928

**Published:** 2023-04-27

**Authors:** Gi-Deok Eom, Ki-Back Chu, Hae-Ji Kang, Min-Ju Kim, Keon-Woong Yoon, Jie Mao, Su-Hwa Lee, Md Atique Ahmed, Eun-Kyung Moon, Fu-Shi Quan

**Affiliations:** 1 Department of Biomedical Science, Graduate School, Kyung Hee University, Seoul, Korea; 2 Medical Research Center for Bioreaction to Reactive Oxygen Species and Biomedical Science Institute, School of Medicine, Graduate School, Kyung Hee University, Seoul, Korea; 3 Department of Medical Zoology, School of Medicine, Kyung Hee University, Seoul, Korea; 4 ICMR-Regional Medical Research Centre, NE Region, Dibrugarh, Assam, India; Instituto Butantan, BRAZIL

## Abstract

*Toxoplasma gondii* host cellular invasion factors such as the rhoptry proteins, micronemal antigens, or other subcellular compartment proteins have shown limited vaccine efficacies. *T*. *gondii* cyst wall protein (CST1) as a cyst persistence factor is critical for cyst wall integrity and bradyzoite persistence. Here, we generated influenza virus-like particles (VLPs) expressing the *T*. *gondii* CST1 and evaluated the mucosal as well as systemic immunities induced by VLPs. Intranasal immunization with the VLPs induced parasite-specific IgG and IgA antibody responses in sera and intestines. VLP immunization showed higher levels of germinal center B cell response and antibody-secreting cell (ASC) response upon challenge infection, indicating memory B cell response was induced. VLP-immunized mice showed a significant reduction of cyst counts and lower levels of pro-inflammatory cytokines (IFN-γ, IL-6) production in the brain upon *T*. *gondii* ME49 challenge infection compared to unimmunized control. Thus, VLP immunization protected mice from the lethal dose challenge infection with *T*. *gondii* ME49 and did not incur bodyweight loss. These results indicated that *T*. *gondii* CST1 containing VLPs can induce mucosal and systemic immunity and also suggest its developmental potential as an effective vaccine candidate against *T*. *gondii* infection.

## Introduction

*Toxoplasma gondii* is an obligatory intracellular parasite capable of infecting a wide array of vertebrate animals, including humans due to their broad host range [1]. Currently, approximately a third of the entire world’s population is estimated to be affected by this pathogen [2]. *T*. *gondii* infection in humans primarily occurs through ingestion of cyst-contaminated food products. Once inside humans or other intermediate hosts, *T*. *gondii* can thrive as a rapidly replicating infective form known as tachyzoite [2]. The tachyzoite stage can have fatal consequences, particularly in pregnant women, the elderly, and other immunocompromised individuals. These tachyzoites can differentiate into slow-growing bradyzoites in tissues such as the brain. While bradyzoites were often regarded as a benign chronic form of the parasite, their presence was later identified to be associated with several neuropsychiatric disorders such as schizophrenia [3, 4]. Anti-parasitic drugs such as sulfadiazine are capable of reducing the *T*. *gondii* tachyzoite burden in hosts. However, the drugs are ineffective against bradyzoites and pharmacological parameter issues such as low drug tolerance or side effects in patients render their use somewhat undesirable [5]. Vaccines, in stark contrast to drugs, are much safer and do not pose the risk of drug resistance development in pathogens. Furthermore, ecological impacts arising from residual drug release are not an issue with vaccines [6]. As such, there is a growing need to develop toxoplasmosis vaccines that can limit parasitemia and tissue cyst formation.

To date, a wide variety of antigens associated with the cyst wall have been identified and used as vaccine antigens or potential diagnostic tools. For instance, a large number of proteins belonging to the dense granule protein (GRA) family have been used as experimental vaccines [7]. Other members such as the cyst matrix antigen (MAG) families have been used as diagnostic genes for detecting toxoplasmosis in peripheral blood mononuclear cells [8, 9]. *T*. *gondii* CST1 is a glycosylated protein component of the parasite’s cyst wall, which was first characterized and described by Zhang et al. [10]. Several other studies reported to date have elucidated multiple features and parasitic functions associated with this protein. Tomita et al. delineated that the mucin domain of the CST1 is integral for normal cyst wall formation and post-translational modifications at this region via two glycosyltransferases are critical for maintaining its biological function [11, 12]. Defects in this gene resulted in abrogated cyst formation, characterized by the leaking of matrix antigens through the fragile cyst walls and into the host cell [13, 14]. Given that genetic knockout of the CST1 gene reduces the structural integrity of the cysts and suppresses their formation in the brains of mice [11], CST1 would be an ideal candidate antigen for *T*. *gondii* vaccines. Recently, using the *T*. *gondii*-positive sera of naturally infected animals and humans, one study has reported that potent humoral responses can be mounted against the cyst wall antigens [15]. Nevertheless, the immunogenicity of these cyst wall proteins and the role of these CST1-specific antibodies in protection remain poorly understood. Therefore, assessing the immunogenicity of such candidate antigens would shed light on improving the current vaccine design strategy.

There are several challenges to developing an efficacious *T*. *gondii* vaccine. Theoretical safety concerns involving live-attenuated vaccines, such as the potential to revert to their virulent form prevent their use in humans [16]. DNA vaccines were reported to be weakly immunogenic, especially in humans and other primates. Subunit vaccines were also reported to lack immunogenicity, hence requiring the use of adjuvants. Moreover, allergic reaction associated with the subunit protein being expressed in another organism was reported [17]. As such, brain cyst inhibition elicited by DNA vaccines expressing the GRA39 [18] or recombinant microneme protein vaccines [19] resulted in roughly 2-fold brain cyst inhibition. Virus-like particles (VLPs) are highly immunogenic vaccine platforms and compared to the two aforementioned vaccine platforms, experimental *T*. *gondii* VLP vaccines inhibited stronger cyst inhibition in the brains of mice [20]. Nevertheless, even the VLP vaccines failed to completely eliminate *T*. *gondii* cysts in mice, thus necessitating the need for further vaccine development. To accommodate this limitation, we focused on using the cyst persistence factor CST1 and assessed the feasibility of enhanced cyst reduction since most of the *T*. *gondii* vaccine studies reported to date emphasized the use of host invasion factors as antigens. Therefore, in this study, we generated recombinant VLPs expressing the CST1 antigen with influenza matrix protein (M1) core protein. In particular, influenza M1 protein was used as their interaction with the cytoplasmic tail in transmembrane domain of an antigen is crucial for proper assembly of VLPs [21–23]. VLP-based vaccines have shown to be safe and efficacious, with several of them already being approved for commercial use by the United States Food and Drug Administration (FDA) [24]. To the best of our knowledge, we first present a CST1 vaccine study investigating the protective efficacy of this antigen using the highly immunogenic VLP vaccine platform. Our findings reveal that CST1 is a promising vaccine antigen candidate possessing potent immunogenicity that could contribute to the complete removal of brain cysts.

## Materials and methods

### Ethics statement

All animal experiments were approved and performed following the guidelines set out by the Kyung Hee University IACUC (permit ID: KHSASP-20-648). All efforts were made to minimize animal suffering and animals reaching the humane intervention point, which was determined to be body weight loss exceeding 20% of its initial value, were considered dead and humanely euthanized in a CO_2_ chamber [25].

### Mice, cells, parasites, and antibodies

Seven-week-old female BALB/c mice were purchased from NARA Biotech (Seoul, South Korea) and maintained in approved facilities under specific-pathogen-free conditions. *Spodoptera frugiperda* (Sf9) cells were cultured in spinner flasks with SF900II media (Invitrogen, Carlsbad, CA, USA) at 27 °C, 140 rpm. *T*. *gondii* ME49 was maintained in BALB/c mice through serial passaging. Horseradish peroxidase (HRP)-conjugated goat anti-mouse IgG and IgA were purchased from Southern Biotech (Birmingham, AL, USA). Serially passaged *Trichinella spiralis* larvae were acquired from mice. Briefly, *T*. *spiralis*-infected murine muscle tissues were digested in pepsin-HCl solution, and larvae were manually counted under the microscope after repeated washing. Mice were infected with 150 *T*. *spiralis* larvae via oral gavage and immune sera were collected at 4 weeks post-infection, which were subsequently used as a negative control.

### Transmembrane domain prediction and cloning of plasmids for the generation of baculoviruses expressing recombinant CST1

Transmembrane topology of the CST1 antigen was predicted *in silico* using Phobius (Stockholm Bioinformatics Center) as previously described [26]. Total RNA was extracted from *T*. *gondii* ME49 cyst with the RNeasy Mini Kit (Qiagen, Valencia, CA, USA). A portion of the CST1 gene (GenBank: XM_002368554.2) was used as the antigen, which is nearly identical to that reported in the study by Deshmukh et al [15]. Because the sequence used in the aforementioned study did not contain the CST1 transmembrane (TM) domain near the N-terminus, which is crucial for VLP assembly, we incorporated the sequences corresponding to the TM region by cloning the gene from the beginning of its coding sequence (nucleotide positions 37 to 1227). This sequence was PCR-amplified with primers (forward: 5’- ATATGAATTCATGACTGCTCCTTTTTTGAGAGTG -3’; reverse: 5’- ATATAAGCTTTCAAATATCCAGTATTAACGCAGCA -3’) for cloning into the pFastBac vector (Invitrogen, Carlsbad, CA, USA) containing the EcoRI/HindIII restriction enzyme sites. Integration of the CST1 gene into the pFastBac vector was confirmed by restriction enzyme digestion and DNA sequencing. Successful clones were further transformed into DH10Bac competent cells. Single colonies cultured on the LB medium containing 50 μg/ml kanamycin, 10 μg/ml tetracycline, and 7 μg/ml gentamycin were subjected to colony PCR using the M13 primer sets (forward: 5’- GTTTTCCCAGTCACGAC -3’; reverse: 5’- CAGGAAACAGCTATGA-3’) to screen for successful constructs. The influenza matrix protein 1 (M1) gene was cloned as previously described [27]. Recombinant bacmid DNA was extracted from the DH10Bac cells using Plasmid SV Kit (GeneAll Biotechnology, Seoul, Korea), and stored at –20 °C until used.

### Generation of recombinant baculovirus (rBV) and VLPs

Recombinant bacmid DNA was transfected into Sf9 cells with Cellfectin II reagent (Invitrogen, Carlsbad, CA, USA) following the manufacturer’s instructions. To generate VLPs containing CST1 and M1, Sf9 cells were co-infected with rBVs expressing either CST1 or M1 as described previously [28]. Sf9 cells at 2.5 x 10^6^ cells/ml concentration were co-infected with rBVs expressing CST1 and M1 antigens at a ratio of 3:1, 0.1 MOI, and cultured at 27 °C, 140 rpm for 3 days. Centrifugation was performed at 6,000 rpm, 4 °C, 30 min, to remove cells and debris. Supernatants containing VLPs were pelleted by high-speed centrifugation at 30,000 rpm for 30 min at 4 °C. VLPs were purified using the 20-30-60% sucrose density gradient method as described previously [29] and resuspended in PBS. VLPs concentration was measured using the QuantiPro BCA Assay Kit (Sigma-Aldrich, St Louis, MO, USA) and stored at 4°C until use.

### Characterization of the VLPs

VLPs were characterized by transmission electron microscopy (TEM) and western blotting. VLP surfaces were negatively stained with 1.5% phosphotungstic acid (pH, 7.0) prior to TEM imaging. Sodium dodecyl sulfate-polyacrylamide gel electrophoresis (SDS-PAGE) was performed and gels were stained with Coomassie blue. Briefly, Sf9 cells at a density of 2.5 x 10^6^ cells/ml were infected with 0.1 MOI of CST1-rBVs. After 48 h, Sf9 cells were centrifuged at 3,000 rpm for 5 min. and supernatants were harvested. Cells were rinsed twice with PBS and protein lysis buffer was added (PRO-PREP^TM^ Protein Extraction Solution, iNtRON Biotechnology, South Korea). After lysing the cells, they were centrifuged at 13,000 rpm for 5 min and supernatants were carefully collected. VLPs were further characterized via western blotting. After resolving the proteins via SDS-PAGE, they were transferred to a nitrocellulose membrane. Membranes were blocked for 30 min at room temperature using 5% skim milk and washed three times with TBST for 10 min at RT. Sera collected from mice infected with *T*. *gondii* ME49 were used as primary antibodies for CST1 protein (1:500 dilution) and membranes were incubated overnight at 4 °C. Monoclonal M1 antibodies were used to detect M1 protein expressions. The next day, membranes were washed three times with TBST for 10 min at RT and incubated with horseradish peroxidase (HRP)-conjugated anti-mouse IgG secondary antibodies (1:2,000 dilution) at RT for 1 hr. After washing with TBST, protein expressions were detected by ChemiDoc Imaging System (Bio-Rad, Hercules, CA, USA) with enhanced chemiluminescence (ECL).

### Immunization and challenge infection

For immunization, a total of 18 BALB/c mice were evenly divided into the following three groups (n = 6 per group): unvaccinated and uninfected group (Naïve), unvaccinated and infected group (Naïve + Cha), and vaccinated and challenge-infected group (CST1 VLPs). For CST1 VLP immunization, 50 μg of VLPs in 50 μl PBS were administered through the intranasal (IN) route, in which the concentration of the CST1 antigens expressed in the 50 μg of VLPs was 5 μg [30]. Four weeks after prime immunization, mice were boost-immunized with an identical dose via IN route. Four weeks after the boost immunization, mice were orally challenge-infected with 450 *T*. *gondii* ME49 cysts as previously described [31]. Briefly, the brain tissues of *T*. *gondii*-infected mice were collected and homogenized. Cysts were separated from the brain homogenates using the Percoll density gradient. After repeated washing with PBS, purified cysts were resuspended in PBS and subsequently used for infection.

### *T*. *gondii* ME49-specific IgG and IgA antibody responses

Three weeks after each immunization, blood samples of individual mice were collected by retro-orbital plexus puncture and centrifuged at 6,000 rpm, 10 min, 4 °C for serum acquisition. At 40 days post-infection (dpi), mice were sacrificed and intestines were acquired as previously described [31]. Duodenums of mice were longitudinally cut and immersed in 500 μl of PBS. Samples were incubated for 1 hr at 37 °C and centrifuged at 5,000 rpm, 10 min for supernatant collection. *T*. *gondii* ME49-specific antibody responses from sera and intestines were determined using enzyme-linked immunosorbent assay (ELISA) as previously described [20]. For *T*. *gondii* ME49 soluble lysate antigen (Tg ME49 Ag) acquisition, purified cysts from the brain homogenates of mice were sonicated and centrifuged. The supernatant fraction containing the soluble antigens was collected and stored at -20 °C until use. Commercialized *T*. *gondii* antigen purchased from MyBioSource (Cat #: MBS568631, San Diego, CA, USA) was also used as a control. Briefly, flat-bottom 96-well microtitration plates (SPL Life Sciences, Pocheon, Korea) were coated with 4 μg/well of either Tg ME49 Ag or commercialized *T*. *gondii* antigen in 0.05 M, pH 9.6 carbonate-bicarbonate buffer overnight at 4 °C. After washing 3 times with PBS containing 0.05% Tween-20 (PBST) (Sigma Aldrich, St Louis, USA), wells were blocked using 0.2% gelatin in PBST at RT for 30 min. After washing with PBST, diluted sera (1:50 in PBS) were inoculated into each well, and plates were incubated for 1 hr, 37 °C. Plates were incubated for another 1 h at 37 °C after adding horseradish peroxidase (HRP)-conjugated goat anti-mouse IgG and IgA secondary antibodies (1:2,000 dilution in PBS) to respective wells. O-phenylenediamine (OPD) substrate purchased from Sigma-Aldrich (St. Louis, MO, USA) was dissolved in citrate substrate buffer with H_2_O_2_ and 100 μl of this mixture was added to each well. Reactions were stopped by adding 2N H_2_SO_4_ and optical density (OD) values at 450nm were measured using a microplate reader (EZ Read 400, Biochrom Ltd., Cambridge, UK). Mean OD values of each group were determined by calculating the average of all the OD values in the group.

### Antibody-secreting cell (ASC) responses

At 40 dpi, mice were sacrificed and spleens were collected. Spleens were homogenized to prepare single cell suspensions of splenocytes, which were counted under the microscope using a hemocytometer as previously described [31]. Tg ME49 Ag were used to coat the 96-well cell culture plates at a concentration of 4 μg/ml in 0.05 M, pH 9.6 carbonate bicarbonate buffer overnight at 4 °C. After blocking the wells with 0.2% gelatin for 30 min at RT, 1 x 10^6^ splenocytes were seeded into individual wells and cultured using RPMI media supplemented with 10% FBS and 1% penicillin/streptomycin for 5 days at 37 °C. After incubation, supernatants were removed and HRP-conjugated anti-mouse IgG was inoculated into each well. OPD substrates dissolved in citrate buffer were added to the wells and OD_450_ values were measured using a microplate reader.

### Germinal center B (GC B) cells responses by flow cytometry

Splenocytes were prepared for flow cytometric analysis as previously described [32]. Splenocytes were stimulated with Tg ME49 Ag (4 μg/mL) for 2 hr at 37 °C with 5% CO_2_. After antigen stimulation, cells were stained with fluorophore-conjugated GL7 (PE) and B220 (FITC) antibodies purchased from BD Biosciences (Franklin Lakes, NJ, USA) and Invitrogen (Waltham, MA, USA). All flow cytometry procedures were performed according to the manufacturer’s protocol. Stained samples were analyzed using the Accuri C6 flow cytometer and the C6 Accuri software (BD Biosciences, Franklin Lakes, NJ, USA).

### Pro-inflammatory cytokine assays and cyst burden measurement

At 40 dpi, brain tissues of individual mice were collected and homogenized in 500 μl of PBS. After centrifugation, supernatants were carefully removed and stored at -80 °C for cytokine assay. Sedimented pellets were resuspended in 200 μl PBS and subsequently used to quantify the brain cyst burden. Production of the pro-inflammatory cytokines IFN-γ and IL-6 was assessed using BD OptEIA ELISA kits (BD Biosciences, Franklin Lakes, NJ, USA) following the manufacturer’s instructions. For brain cyst count, 5 μl of the cyst suspension was placed on top of a clean slide glass and cysts were counted under the microscope.

### Immunofluorescence assay (IFA)

All experimental procedures were carried out at room temperature. Briefly, cysts were isolated from the brains of *T*. *gondii* ME49-infected mice at 8 weeks post-infection. After purifying the cysts, they were carefully placed on coverslips and fixed with 4% paraformaldehyde (PFA) for 10 min and subsequently blocked with 2% BSA dissolved in PBS. After washing the coverslips thrice with PBS, they were incubated with the polyclonal CST1 immune sera acquired from mice (1:100 dilution in PBS). Coverslips were washed and incubated with FITC-conjugated anti-mouse IgG antibody (Median Diagnostics, ChunCheon, Korea; 1:300 dilution in PBS) for 1 h. After the final washing step, coverslips were mounted using a mounting medium (Vector Laboratories Inc., Burlingame, CA, USA). Slides were observed using a fluorescent microscope (Leica DMi8, Wetzlar, Germany) and all images were acquired at 400x magnification.

### Bodyweight and survival measurement

Mice were monitored daily to record bodyweight changes and survival. At 40 dpi, which corresponds to the humane intervention point as all of the mice in the infection control group had bodyweight loss exceeding 20%, all of the mice were sacrificed.

### Statistical analysis

All statistical analyses were performed using the GraphPad Prism 8 software (San Diego, CA, USA). All samples were collected on an individual basis and each experiment was performed 3 times in triplicates. Representative data from two independent vaccination and challenge infection studies were used. Data were expressed as mean ± SD and statistical significances between the means of groups were determined via Student’s *t*-test or one-way ANOVA test followed by Dunnet’s or Tukey’s *post hoc* test (*P < 0.05, **P < 0.01, ***P < 0.001).

## Result

### CST1 gene sequence verification, cloning, and VLP characterization

**Fig 1:**
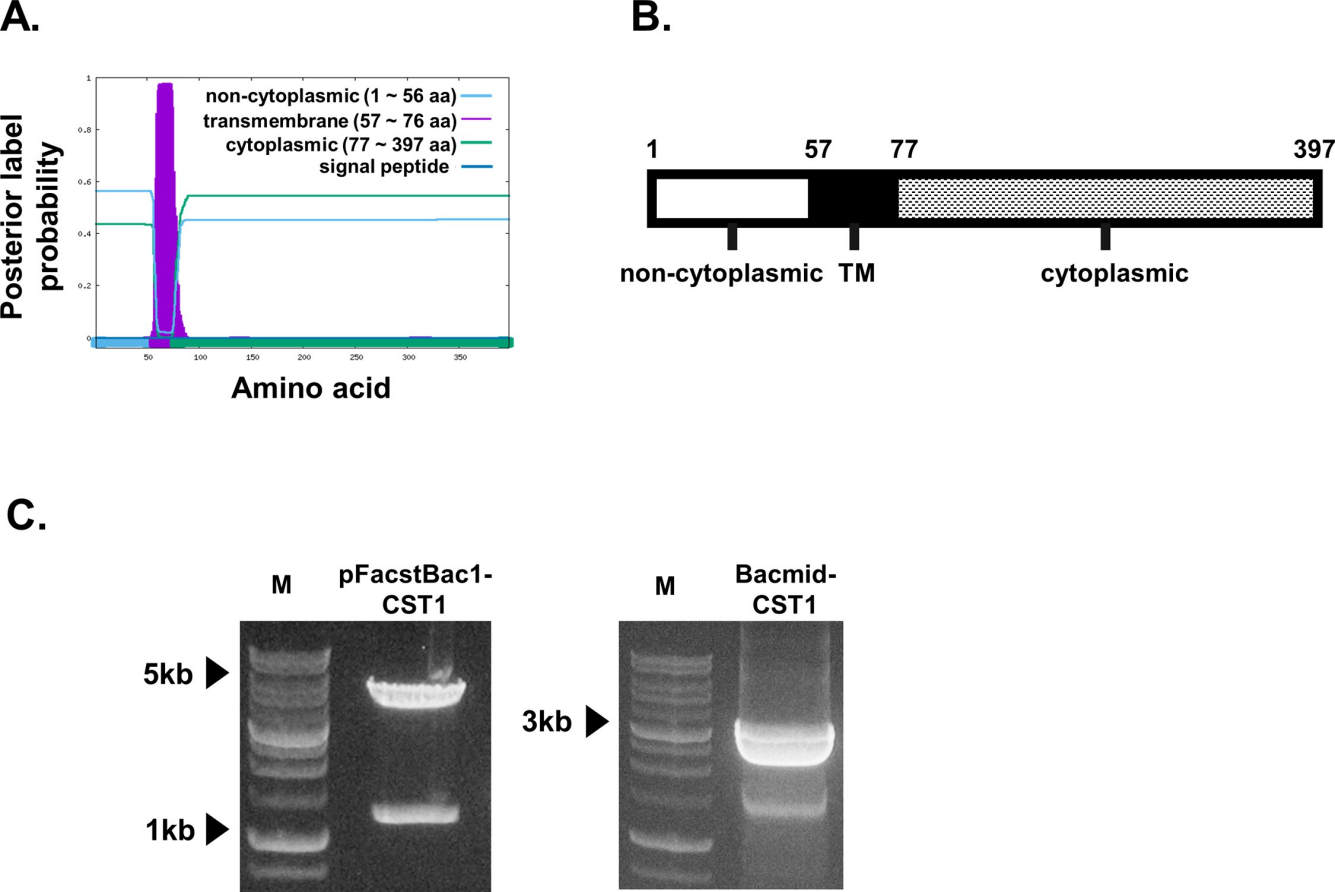
TM prediction, diagram and gene cloning. (A) The transmembrane structure was analyzed by performing an *in silico* analysis of the CST1 protein amino acid sequence with Phobius. The x-axis represents the position of the amino acid, and the y-axis represents the probability that the amino acid at the corresponding position occupies each region or domain. (B) A schematic diagram depicting the CST1 gene used in this study (positions 1–397) was also provided, with the numbers denoting amino acid positions. (C) Recombinant pFastBac1 vector expressing the CST1 gene was digested with restriction enzymes to confirm CST1 gene integration (left panel). Colony PCR was performed to confirm the successful transposition of the CST1 gene into the bacmid DNA (right panel).

**Fig 2:**
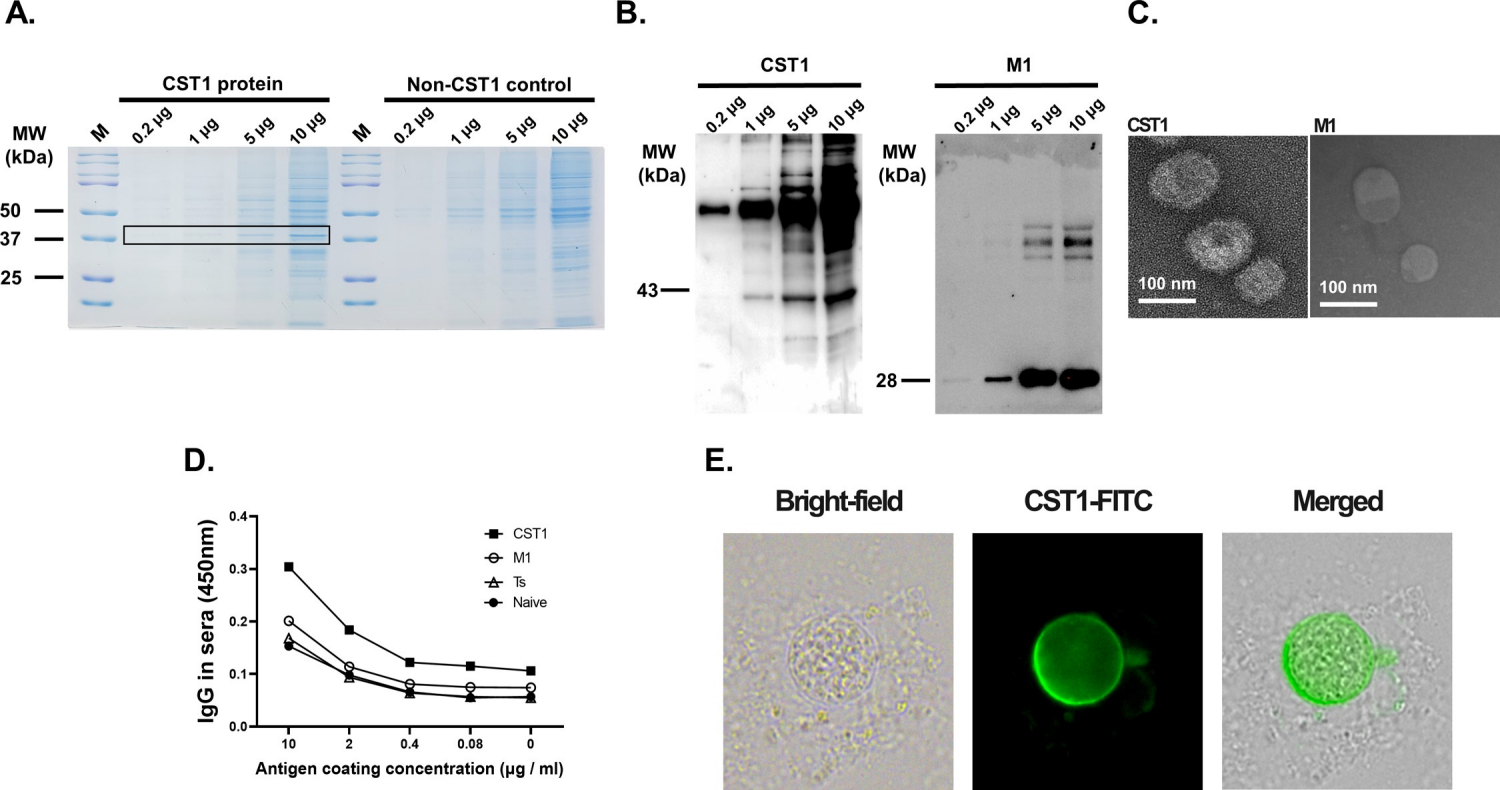
VLPs characterization and immunofluorescence assay. (A) SDS-PAGE was performed using the whole lysates of Sf9 cells which were either infected or uninfected with the CST1-rBVs. Gels were stained with the Coomassie blue dye to identify CST1 protein expression and its separation. The boxed region corresponds to CST1 which has a molecular weight of 43 kDa. (B) Antigen components of the VLPs were characterized using western blot after probing the membranes with polyclonal mouse anti-*T*. *gondii* sera and anti-M1 monoclonal antibody. (C) Morphologies of the purified VLPs and the vehicle control (M1 VLP), which do not express the CST1 gene were examined by transmission electron microscopy. (D) Serum antibody responses against serially diluted concentrations of *T*. *gondii* ME49 were evaluated by ELISA (Ts: *T*. *spiralis* infection sera, M1: M1 VLP immune sera). (E) Immunofluorescence assays were performed to assess whether the polyclonal sera acquired from *T*. *gondii*-infected mice could interact with the CST1 antigens. Images were acquired at 400x magnification under a fluorescent microscope.

Transmembrane topology and signal peptides were predicted using the Phobius web server (Fig 1A). The image illustrated here provides information in the form of probabilities. The x-axis represents the position of the amino acid and the y-axis represents the probability that the amino acid at that position occupies each region or domain. A schematic diagram depicting the CST1 amino acid sequences including TM used in the study was provided (Fig 1B). Successful cloning of the CST1 gene into the pFastBac1 vector was confirmed using restriction enzyme digestion with EcoRI and HindIII, with bands indicating the pFastBac1 vector and CST1 being detected at 4.8 kbp and 1.2 kbp, respectively (Fig 1C, left). The pFastBac1-CST1 construct was transformed into the DH10Bac competent cell and successful integration of the CST1 gene was confirmed by colony PCR (Fig 1C, right). After VLP vaccine production, CST1 VLPs were further characterized using western blot and TEM. Separation of CST1 protein was identified by Coomassie blue staining of the SDS-PAGE gel (Fig 2A). Distinct bands corresponding to the CST1 antigens were only observed from Sf9 cells infected with the CST1 rBVs, but not from uninfected Sf9 control cells. Bands indicating the expressions of CST1 and M1 antigens were observed at 43 kDa and 28 kDa, respectively (Fig 2B). Under the TEM, the size of the VLPs was estimated to be approximately 100 nm (Fig 2C, left). CST1 protein expression on the VLP surface was also visible. M1 VLPs, which clearly lack the CST1 antigen spikes expressed on the surface were also visualized using TEM (Fig 2C, right). To demonstrate that M1 protein in the VLPs does not contribute to parasite-specific antibody responses, ELISA was confirmed. Incremental increases in antibody responses were elicited by *T*. *spiralis* and M1 immune sera against the *T*. *gondii* ME49 lysate antigens, but the overall changes were negligible (Fig 2D). To further confirm that the sera acquired from CST1 VLP-immunized mice were capable of detecting CST1 in the cysts, immunofluorescence assays were performed. Round cysts with intact cyst walls were visualized under bright-field microscopy. Using FITC-conjugated secondary antibodies, CST1 VLP immune sera interaction with the cyst wall was confirmed (Fig 2E).

### *T*. *gondii* ME49-specific IgG and IgA antibody responses in sera

**Fig 3:**
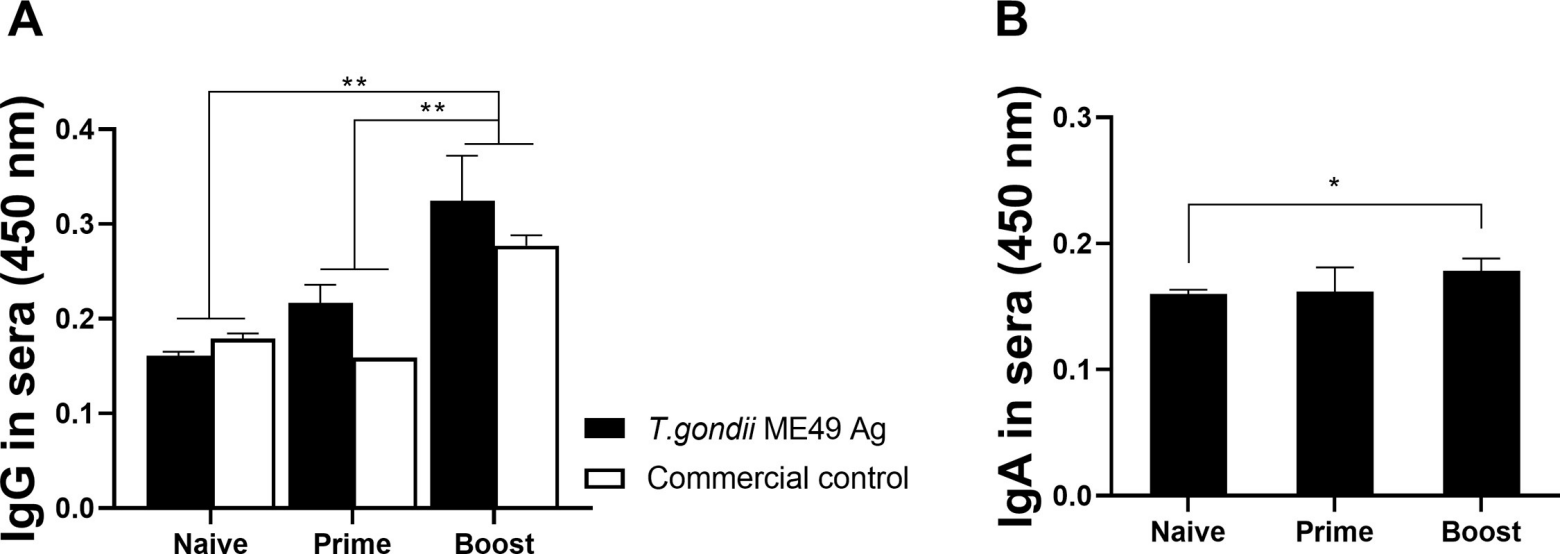
IgG and IgA in sera. Mice were intranasally immunized twice with CST1 VLPs at a 4-week interval and sera were collected 3 weeks after each immunization. *T*. *gondii*-specific IgG (A) and IgA (B) antibody responses from the sera were measured using ELISA. Absorbance values at 450nm were measured and data are shown as mean ± SD, with asterisks denoting statistical significance between the groups (*P < 0.05, ***P < 0.001). Serum samples were collected on an individual basis and data are representative of 3 independent experiments performed in triplicates.

Mice were immunized twice with the CST1 VLP vaccine through the intranasal route at 4-week intervals, and sera were collected 3 weeks after each immunization. *T*. *gondii* ME49-specific IgG and IgA antibody responses from sera were evaluated by ELISA. Compared to the unimmunized control, *T*. *gondii* ME49-specific IgG responses from the sera of CST1 VLP-immunized mice were increased after prime immunization. This was further enhanced after boost immunization, with significant differences being observed compared to both naïve control and prime immunization response. Overall, *T*. *gondii*-specific serum IgG responses elicited against the commercialized *T*. *gondii* antigen were comparable to those induced against Tg ME49 (Fig 3A). *T*. *gondii* ME49-specific IgA responses after priming with the CST1 VLPs were similar to that of naïve control mice. Boost immunization elicited marginally elevated parasite-specific IgA responses which were significantly greater than those of naïve sera (Fig 3B). To evaluate whether the *T*. *gondii* ME49 immune sera were truly CST1-specific, IFA was performed (Fig 2E). Fluorescence was detected from the entirety of the cyst surface, thus confirming that the antibody used in the study was capable of detecting the CST1 antigen that lines the cyst walls.

### *T*. *gondii* ME49-specific intestinal IgG and IgA antibody responses

**Fig 4:**
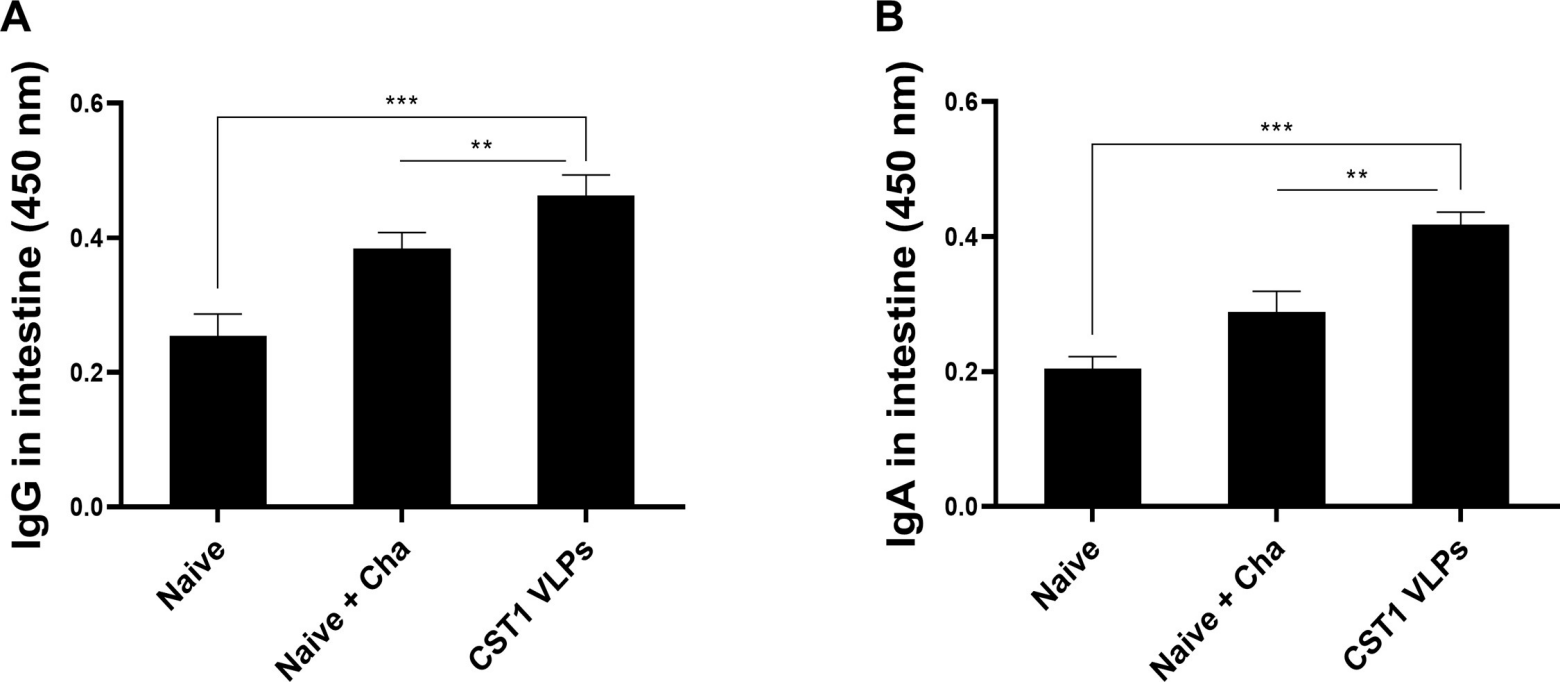
IgG and IgA in the intestine. Intestines of mice were collected after challenge infection with *T*. *gondii* ME49. Mucosal IgG (A) and IgA (B) antibody responses from the intestines were measured by ELISA. Absorbance values at 450nm were measured and data are shown as mean ± SD, with asterisks denoting statistical significance between the groups (**P < 0.01, ***P < 0.001). Mucosal samples were collected on an individual basis and data are representative of 3 independent experiments performed in triplicates.

At 40 dpi, mice were sacrificed and intestines were collected to assess *T*. *gondii* ME49-specific mucosal antibody response induction. Parasite-specific intestinal IgG responses were significantly higher in the immunized mice post-challenge infection compared to naïve control (Fig 4A). Significant differences were also observed between Naïve + Cha and CST1 VLP-immunized mice. This was also the case for intestinal IgA, with VLP immunization eliciting higher mucosal antibody production (Fig 4B). Findings presented here revealed that IN immunization with the CST1 VLPs is capable of eliciting mucosal antibody responses that may contribute to protection.

### CST1 VLP immunization induces germinal center B cell response

**Fig 5:**
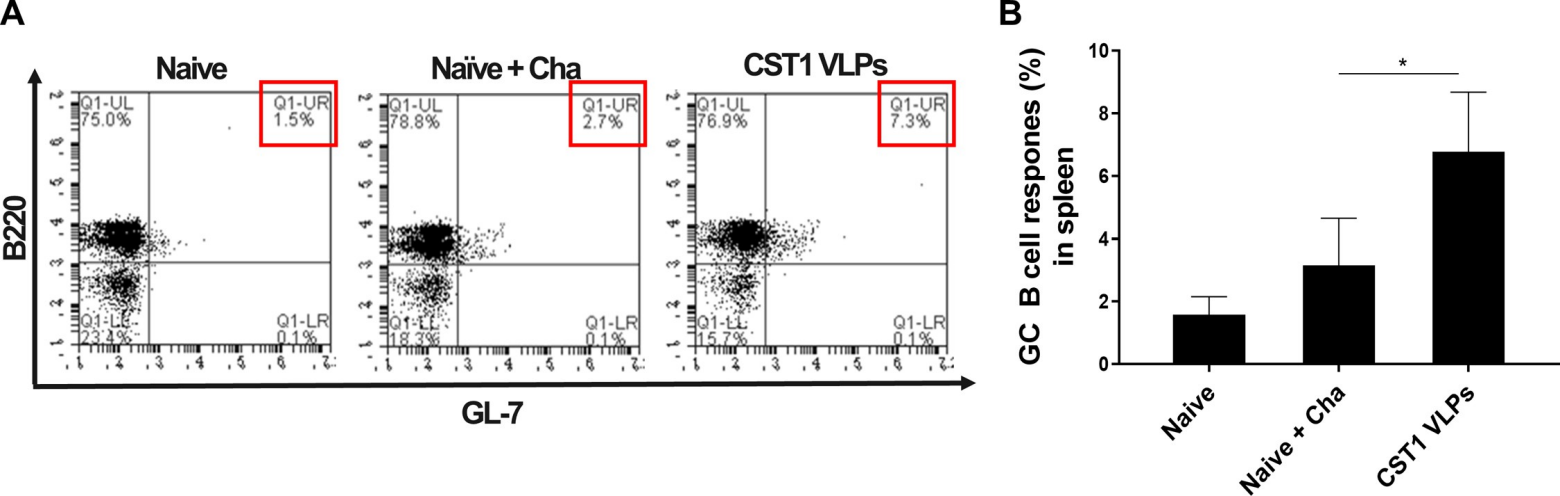
Germinal center B cell responses in spleen. BALB/c mice were intranasally immunized twice with CST1 VLPs at a 4-week interval. Four weeks after the final immunization, mice were orally challenge-infected with *T*. *gondii* ME49. Spleens were harvested at 40 dpi and single cell splenocyte populations were stimulated with the Tg ME49 Ag. GC B cell populations were determined using flow cytometry. (A) Representative scatter plots showing B220+/GL-7+ GC B cells from each group were illustrated. (B) Mean GC B cell responses from each group were depicted using a bar graph. Data are shown as mean ± SD, and an asterisk was used to denote statistical significance between the groups (*P < 0.05). Splenocytes were collected on an individual basis and representative flow cytometry data acquired from 2 independent vaccination and challenge infection studies were used.

Murine splenocytes were acquired at 40 dpi and GC B cell propagation was evaluated using flow cytometry. Compared to the naive control (1.5%), as demonstrated in the representative flow cytometry plots, infecting unimmunized mice with the *T*. *gondii* ME49 marginally elevated the GC B cell population to 2.7% (Fig 5A). However, in the splenocytes of CST1 VLP-immunized mice, this value was significantly elevated to 7.3%. The mean values of the GC B cell populations also followed this trend, with significant differences being observed between Naïve + Cha and CST1 VLP groups (Fig 5B).

### Antibody-secreting cell responses in the spleen

**Fig 6:**
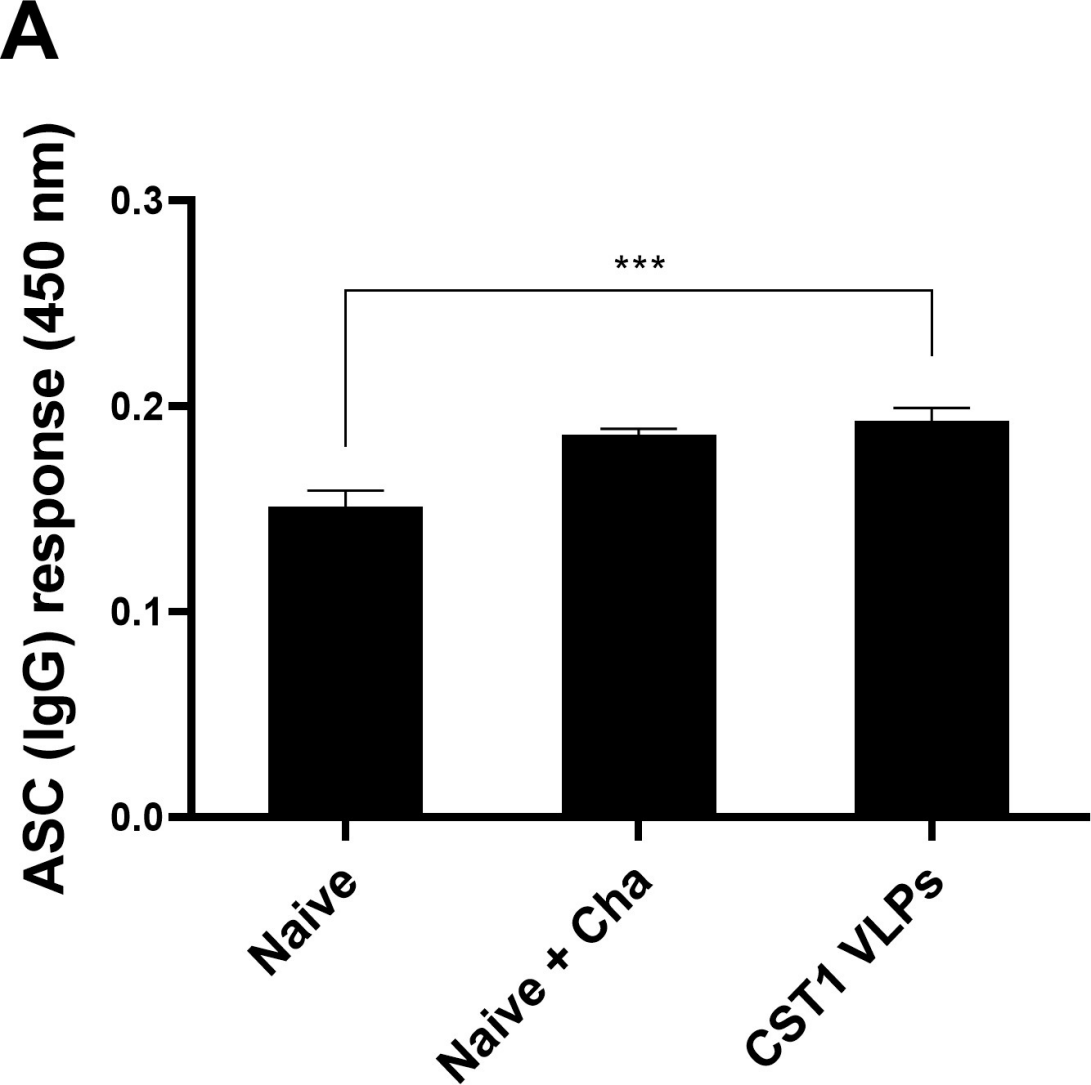
Splenic ASC responses. Splenocytes of individual mice were acquired at 40 dpi and seeded into 96 well plates coated with Tg ME49 Ag. *T*. *gondii*-specific IgG antibody-secreting cell response was determined by ELISA. Absorbance values at 450nm were measured and data are shown as mean ± SD, with asterisks denoting statistical significance between the groups (***P < 0.001). Splenocytes were collected on an individual basis and representative flow cytometry data acquired from 2 independent vaccination and challenge infection studies were used.

To measure *T*. *gondii* ME49-specific IgG antibody-secreting cell response, murine splenocytes were cultured for 5 days in 96 well plates coated with Tg ME49 Ag. The ASC IgG response elicited by CST1 VLP immunization was significantly different compared to naive control, but no difference was observed between CST1 VLPs and Naïve+Cha (Fig 6).

### Pro-inflammatory cytokine responses in the brain

**Fig 7:**
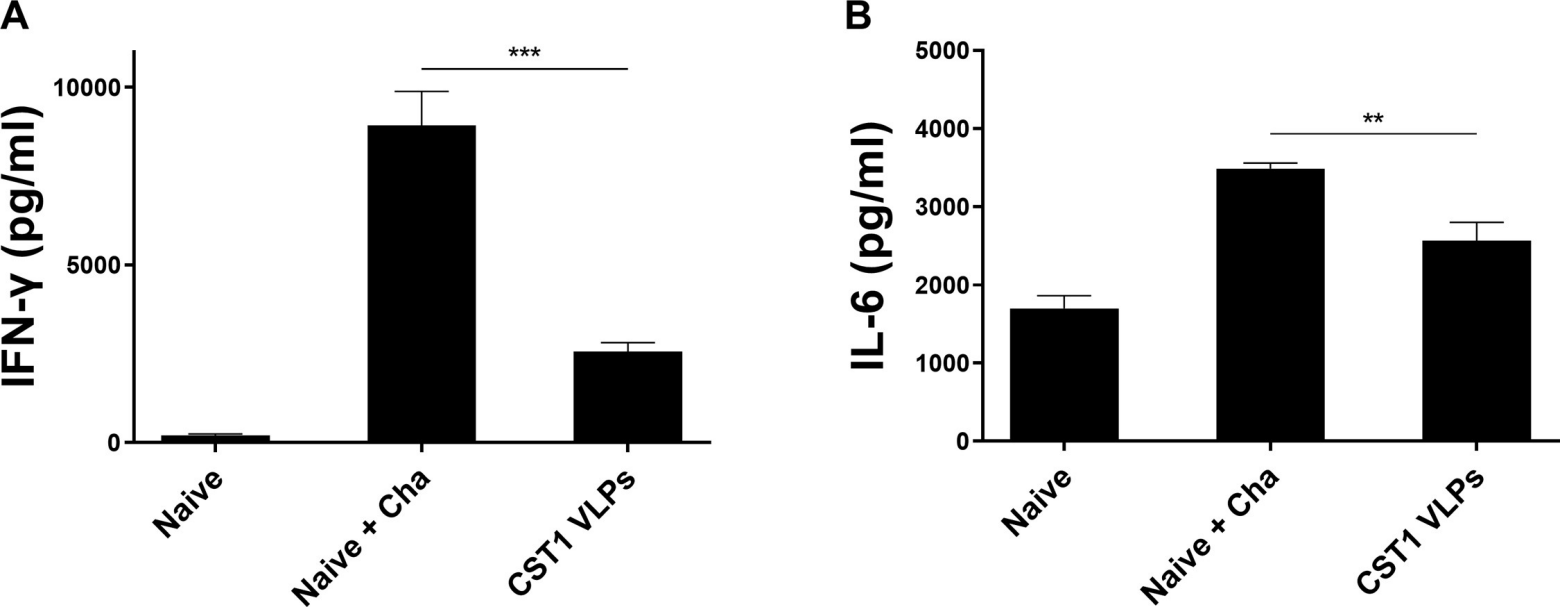
Pro-inflammatory cytokine responses in the brain. Brain tissues of mice were harvested at 40 dpi with *T*. *gondii* ME49 to assess the production of pro-inflammatory cytokines. Supernatants of brain homogenates were used to assess (A) IFN-γ and (B) IL-6. Data are shown as mean ± SD, and asterisks denote statistical significance between the groups (**P < 0.01, ***P < 0.001). Tissue samples were collected on an individual basis and data are representative of 3 independent experiments performed in triplicates.

To assess the anti-inflammatory response induced by immunization, pro-inflammatory cytokines IFN-γ and IL-6 productions were measured. Compared to the Naïve + Cha group, CST1 VLP immunization reduced the production of IFN-γ by nearly 3-fold in the brains of mice (Fig 7A). A similar trend was also observed for IL-6 responses, although VLP vaccine-induced cytokine reductions were not as drastic (Fig 7B). Through these results, increased production of pro-inflammatory cytokines after *T*. *gondii* ME49 infection was confirmed. However, CST1 VLP immunization mitigated the pro-inflammatory cytokine production in the brains of mice.

### Protective efficacy of CST1-VLP immunization against ME49 challenge infection

**Fig 8:**
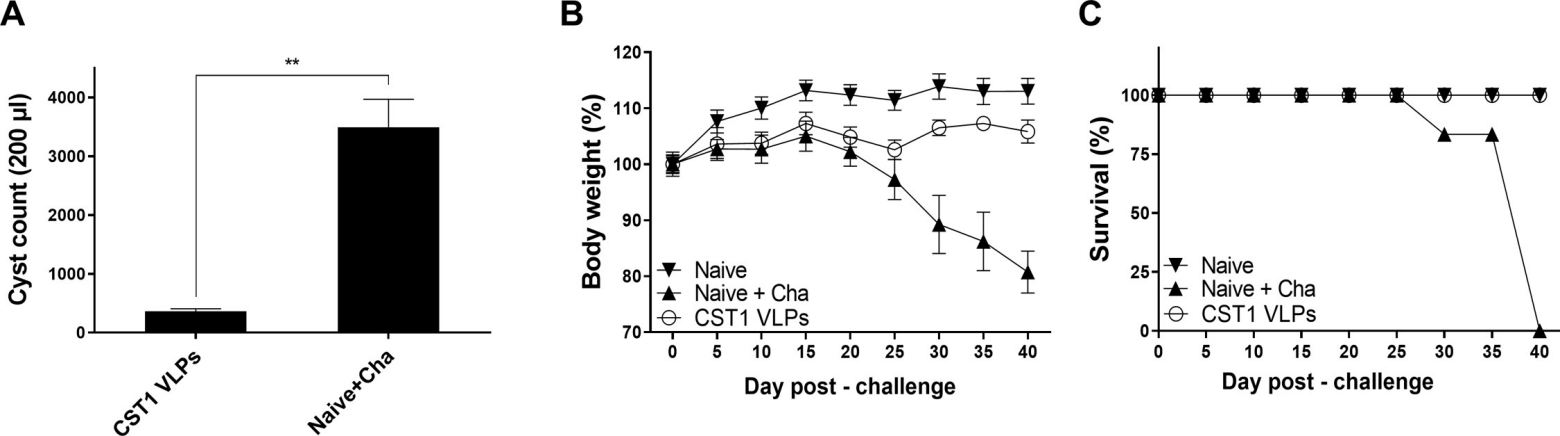
Cyst count, bodyweight and survival. BALB/c mice were orally challenge-infected with a lethal dose of *T*. *gondii* 4 weeks after the boost immunization. Brain tissues were harvested at 40 dpi and (A) cysts were enumerated after isolation from brain homogenates. (B) Changes in bodyweight and (C) survival rates were assessed daily after challenge infection. Data are shown as mean ± SD, and asterisks denote statistical significance between the groups (**P < 0.01). Tissue samples were collected on an individual basis and representative data acquired from 2 independent vaccination and challenge infection studies were used.

To determine the protective efficacy of CST1 VLP vaccines, both immunized and unimmunized mice were challenge-infected with a lethal dose of *T*. *gondii* ME49. At 40 dpi, diminished brain cyst counts were observed in the VLP-immunized mice (Fig 8A; **P < 0.01). Consistent with this finding, drastic bodyweight changes were not observed in CST1 VLP-immunized mice. However, bodyweight reductions in the Naïve + Cha became noticeable from 25 dpi onward. By 40 dpi, the bodyweights of Naïve + Cha mice reached the humane intervention point and all of the remaining animals were humanely euthanized (Fig 8B). At 30 dpi, a mouse in the Naïve + Cha group perished (Fig 8C). By 40 dpi, the remaining mice in the Naïve + Cha group had to be euthanized whereas 100% survival was observed in naïve and CST1 VLP immunization groups.

## Discussion

VLPs are highly sought after for their potential as vaccines. In these vaccine platforms, target antigens are arranged into densely packed highly repetitive elements that promote mounting of efficient antibody response [33, 34]. Their small particulate nature favors uptake by antigen-presenting cells and ultimately contributes to stimulating both the innate and the adaptive immune responses [35]. Furthermore, because VLP vaccines lack the genetic materials required for replication, they are non-pathogenic and inherently safe for use [36]. Here, we generated VLPs expressing the *T*. *gondii* CST1 antigen using the influenza M1 via baculovirus expression system. Our findings demonstrated that the CST1 VLP vaccines were highly immunogenic and successfully protected mice against lethal challenge infection with the *T*. *gondii* ME49 strain.

Immunofluorescence assays (IFA) from previous studies have demonstrated that the staining patterns substantially differ between *in vitro*-induced bradyzoites and intracellular tachyzoites [37]. Consistent with this finding, another study confirmed the distinct expression of CST1 antigens on the outer surface of *T*. *gondii* bradyzoites [38, 39]. Here, our IFA demonstrated that CST1 antigens are expressed throughout the entire surface of the *T*. *gondii* cysts. Our CST1 immunofluorescence results were similar to the initial characterization data previously reported by Zhang et al. [10]. Given the presence of CST1 antigens in multiple stages of the *T*. *gondii* life cycle, CST1 is thought to be a potential vaccine candidate. Intranasal immunization with the CST1 VLPs elicited mucosal immune responses in the intestines. This was expected since both gut-associated lymphoid tissue (GALT) and nasopharyngeal-associated lymphoid tissue (NALT) are part of the mucosa-associated lymphoid tissue (MALT) network. While mucosal immunity is generated in the inductive sites, which would be NALT in this study given the nature of IN immunization, it is well established that antigen-specific mucosal IgA responses can be elicited at different effector sites [40, 41]. This interconnected network of common mucosal immune system enabled the *T*. *gondii*-specific IgA responses to be observed from the intestinal tissues, despite the mice being immunized through IN route in our study. In support of this notion, our previous findings also demonstrated that intestinal antigen-specific antibody responses can be evoked through intranasal immunization [32, 42].

B cells are essential for mounting protective immunity against *T*. *gondii*, as these contribute to producing antibodies that deter tachyzoites from infecting the host cells [43, 44]. Intranasal administration of CST1 VLPs into mice significantly enhanced GC B cell propagation and evoked *T*. *gondii*-specific antibody responses from various organs. Previously, sera acquired from *T*. *gondii*-infected individuals or animals were reported to elicit a stronger antibody response to the CST1 antigen than the bradyzoite surface antigen, which could be attributed to enhanced CST1 antigen exposure to the host’s immune system as the cysts rupture [15]. Based on this finding, we anticipated that CST1 antigens were highly immunogenic and would enable potent antibody response induction. These results were further supported by the protection induced by the CST1 VLPs. Previously, we have assessed the protective efficacies of several VLP-based vaccines against the *T*. *gondii* ME49 strain, which includes VLPs co-expressing the inner membrane complex (IMC), rhoptry protein 18 (ROP18), and microneme protein 8 (MIC8) [25, 32, 45]. Other VLPs co-displaying the ROP4 and ROP13 antigens [31, 46] or the MIC8 and ROP18 antigens have been generated and their efficacies were evaluated in mice [20]. Surprisingly, the CST1 VLPs generated in this study demonstrated the strongest brain cyst inhibition, surpassing those reported in our previous works. Interestingly, such results were observed from the CST1 VLPs despite the lesser immunization dose, lesser number of immunizations, and even without adjuvant usage.

Increasing the number of immunizations and the use of adjuvants enhances cyst burden reductions in mice [25, 32]. Previously, we have shown that immunizing mice with the CpG ODN-adjuvanted multi-antigenic VLPs expressing IMC, ROP18, and MIC8 elicits a 4-fold reduction in total brain cyst after 2 immunizations [32]. However, unadjuvanted VLPs solely expressing the CST1 antigen elicited a 10-fold reduction at an identical number of immunizations. Interestingly, in the current study, VLPs solely expressing the CST1 antigen were immunized using half of the dose used in our previous study with a lesser number of immunizations. Despite the absence of adjuvants, CST1 VLP immunization resulted in nearly identical cyst burden reduction reported in the aforementioned studies, which were the result of 3 immunizations. Also, our previous studies demonstrated that combining multiple VLPs or using a VLP co-expressing multiple antigens for immunization elicited a stronger immune response and protection than VLPs displaying a single antigen [28, 45]. Nevertheless, a single CST1 antigen-expressing VLP vaccine conferred a similar degree of protection to those of multi-antigenic VLPs even at a lower immunization dose. IFN-γ, though dispensable for *T*. *gondii* cyst formation in brain cells, is crucial for the survival of cysts as well as for regulating tachyzoite proliferation [47]. IL-6 is another important cytokine whose expression level appears to be negatively correlated with protection. Evidently, failure to mediate IL-6 by suppressor of cytokine signaling molecule 3 (SOCS3) resulted in mice succumbing to acute *T*. *gondii* infection [48]. In this study, mice immunized with the CST1 VLP vaccine had significantly lower expression levels of pro-inflammatory cytokines IFN-γ and IL-6 compared to the non-immunized infected control group. CST1 VLP-vaccinated group had less inflammatory response even when infected with a lethal dose of *T*. *gondii*. The significantly lessened inflammatory responses in mice contributed to reducing cyst burden and ensured 100% survival of mice.

CST1 VLPs demonstrated here conferred potent immune responses that contributed to protection against ME49 challenge infection. Based on this notion, we speculate that this VLP may even be effective against other *T*. *gondii* strains such as the virulent RH strain. Recently, one study evaluated the protective efficacy of DNA vaccines expressing the CST2 antigen in BALB/c mice but the protection induced was limited [49]. Nonetheless, it is indubitable that CST2 is an antigen highly sought after as knocking out these genes completely inhibited cyst formation in the brains of mice [50]. Therefore, incorporating the CST2 antigen into the VLP vaccine used in the present study could further enhance its protective efficacy. Using this concept, co-expressing multiple key antigens reflective of different *T*. *gondii* stages could yield promising results.

In conclusion, we show that *T*. *gondii* CST1 VLPs can induce systemic and mucosal immune responses that confer protection against *T*. *gondii* ME49 challenge infection. While our results showed the potential of CST1 VLPs as a *T*. *gondii* vaccine candidate, further efforts to improve their efficacy by incorporating CST1 antigen into multi-antigenic vaccines or the use of adjuvants should be warranted. The protection elicited by these vaccines should also be evaluated against multiple *T*. *gondii* strains in other animal models. Our findings presented here pave the way to developing an efficacious *T*. *gondii* vaccine and highlight the potential of CST1 antigen as a vaccine candidate.
